# Components of the Nucleotide Salvage Pathway Increase Frog Virus 3 (FV3) Replication

**DOI:** 10.3390/v15081716

**Published:** 2023-08-10

**Authors:** Samantha R. Logan, Mark Seegobin, R. J. Neil Emery, Craig R. Brunetti

**Affiliations:** Department of Biology, Trent University, 1600 West Bank Dr., Peterborough, ON K9J 7B8, Canada; samanthalogan@trentu.ca (S.R.L.); nemery@trentu.ca (R.J.N.E.)

**Keywords:** *Iridoviridae*, frog virus 3, FV3, ranavirus, viral replication, purine salvage, nucleotide metabolism, adenine, adenosine

## Abstract

Viruses are obligate intracellular parasites that alter host metabolic machinery to obtain energy and macromolecules that are pivotal for replication. *Ranavirus*, including the type species of the genus frog virus 3 (FV3), represent an ecologically important group of viruses that infect fish, amphibians, and reptiles. It was established that fatty acid synthesis, glucose, and glutamine metabolism exert roles during iridovirus infections; however, no information exists regarding the role of purine metabolism. In this study, we assessed the impact of exogenously applied purines adenine, adenosine, adenosine 5′-monophosphate (AMP), inosine 5′-monophosphate (IMP), inosine, S-adenosyl-L-homocysteine (SAH), and S-adenosyl-L-methionine (SAM) on FV3 replication. We found that all compounds except for SAH increased FV3 replication in a dose-dependent manner. Of the purines investigated, adenine and adenosine produced the most robust response, increasing FV3 replication by 58% and 51%, respectively. While all compounds except SAH increased FV3 replication, only adenine increased plaque area. This suggests that the stimulatory effect of adenine on FV3 replication is mediated by a mechanism that is at least in part independent from the other compounds investigated. Our results are the first to report a response to exogenously applied purines and may provide insight into the importance of purine metabolism during iridoviral infection.

## 1. Introduction

**Figure 1:**
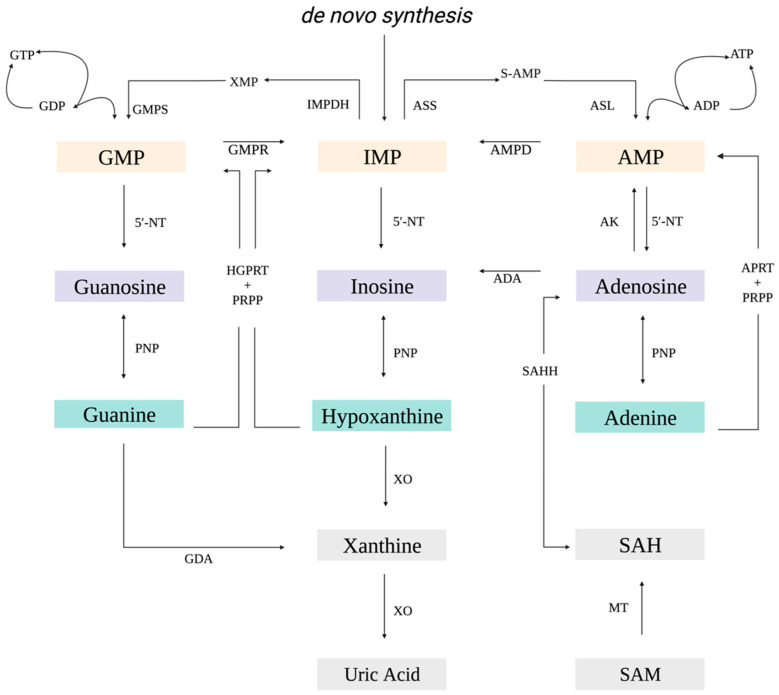
Schematic of purine metabolism. ADA, adenosine deaminase; ADP, adenosine diphosphate; AK, adenosine kinase; AMP, adenosine 5′-monophosphate; AMPD, AMP deaminase; APRT, adenine phosphoribosyltransferase; ASL, adenine succinate lyase; ASS, adenine succinate synthase; ATP, adenosine triphosphate; GDA, guanine deaminase; GDP, guanosine diphosphate; GMP, guanosine 5′-monophosphate; GMPR, guanosine 5’-monophosphate oxidoreductase; GMPS, guanosine 5’-monophosphate oxidoreductase; GTP, guanosine triphosphate; IMP, inosine 5′-monophosphate; HGPRT, hypoxanthine-guanine phosphoribosyltransferase; MT, methyltransferases; 5′-NT, 5′-nucleotidase; PNP, purine nucleotide phosphorylase; PRPP, phosphoribosyl pyrophosphate; SAH, S-adenosyl-L-homocysteine; SAHH, S-adenosyl-L-homocysteine hydrolase; SAM, S-adenosyl-L-methionine; S-AMP, adenylosuccinate; XMP, xanthosine monophosphate; and XO, xanthine oxidase. Orange, purple, and green boxes correspond to nucleotides, nucleosides, and nitrogenous bases, respectively.

Purines represent a class of small molecules essential for a wide variety of cellular processes, including cell signaling, metabolism, and nucleic acid biosynthesis [1,2]. Purine nucleotides are synthesized de novo or recycled through purine salvage. De novo synthesis represents an energetically intensive sequence initiated with the formation of phosphoribosyl pyrophosphate (PRPP) that is converted through ten sequential reactions orchestrated by six enzymes to inosine 5′-monophosphate (IMP; Figure 1) [1,3,4]. In contrast, purine salvage is an energetically favourable process that relies on the by-products of cellular metabolism to interconvert bases, nucleosides, and nucleotides [3,5]. IMP, acting as an intermediary, is converted to either adenosine 5′-monophosphate (AMP) or guanosine 5′-monophosphate (GMP; Figure 1) [1,3,4]. Hypoxanthine-guanine phosphoribosyltransferase (HGPRT) in combination with PRPP catalyzes the conversion of hypoxanthine to IMP or guanine to GMP (Figure 1) [5,6]. Similarly, adenine phosphoribosyltransferase (APRT) converts adenine to AMP (Figure 1) [5]. Adenosine is tightly regulated to maintain adenylate pools and reduce the free adenosine that arises from both nucleic acid catabolism and S-adenosyl-L-methionine (SAM)-dependent methylation reactions [3,7,8]. S-adenosyl-L-homocysteine (SAH), the product of these methylation reactions, is converted in a reversible reaction to adenosine by S-adenosyl-L-homocysteine hydrolase (SAHH; Figure 1). Adenosine is then recycled by adenosine kinase or adenosine deaminase to form AMP and inosine, respectively [3,7,8].

Viruses are obligate intracellular parasites relying on the metabolic system of host cells to provide energy and synthesize the macromolecules necessary for replication. It is well established that viral infections alter aspects of host cell lipid, amino acid, energy, and nucleotide metabolism [9,10,11,12]. While changes to nucleotide metabolism are routinely reported, less is known about the response of viral infections to exogenous application of components of purine salvage. Members of the *Flaviviridae* family show marked reductions in replication upon exposure to guanosine [13] and SAM [14,15]. Exposure of hepatitis C virus (HCV) to guanosine (IC_50_ = 164 μM) increases indel frequencies in HCV DNA by altering intracellular pools of di- and triphosphate ribonucleosides (NDP and NTP), ultimately inhibiting the activity of HCV RNA-dependent RNA polymerase NS5B [13], while SAM (1 mM) reduces HCV NS5A levels by 90% though modulation of antioxidant enzymes and increased glutathione synthesis [14]. Contradictorily, exposure of hepatitis E virus (HEV), an RNA virus of the family *Hepeviridae*, to guanosine (10–100 μg/mL) stimulates viral replication, indicating some involvement of nucleotide biosynthesis in HEV replication [16].

In DNA viruses, exogenous application of purine salvage molecules have been demonstrated as potent antivirals. In vitro application of adenosine (EC_50_ = 0.14 mM) effectively impairs vaccinia virus (VACV) replication via increased cAMP levels and cAMP-dependent protein kinase (PKA) activation, which is mediated by A_2_ receptors [17]. In the *Herpesviridae* family, Epstein–Barr virus (EBV) DNA polymerase is inhibited by GMP [18]. Similarly, herpes simplex virus (HSV) is inhibited by purine salvage molecules both in vitro [19] and in vivo [20]. In vitro, 2 mM of GMP, IMP, or AMP reduces HSV DNA polymerase activity by 47.9%, 49.7%, and 66.6%, respectively [19]. In vivo, application of AMP (EC_50_ = 2.0 mg/kg) prevents the establishment of HSV latency and reduces virus-associated lesion formation [20].

*Iridoviridae* is a family of large (~120–200 nm), nucleocytoplasmic, circularly permutated, and terminally redundant linear double-stranded DNA (dsDNA) viruses with icosahedral symmetry [21]. The *Iridoviridae* family is composed of six genera that infect invertebrate hosts (*Iridovirus*, *Chloriridovirus*, and *Decapodiridovirus*) or ectothermic vertebrates (*Megalocytivirus*, *Lymphocystivirus*, and *Ranavirus*) including bony fish, amphibians, and reptiles [22,23]. Frog virus 3 (FV3), the type species of the genus *Ranavirus*, and other iridoviruses are unique in that both enveloped and non-enveloped virions are infectious [24,25]. Mechanistically, non-enveloped virions release viral DNA into the cytoplasm upon interaction with the plasma membrane, while enveloped virions enter the cell via receptor-mediated endocytosis and travel in non-enveloped form to the nucleus where viral DNA is injected [24,25,26]. In the nucleus, temporal transcription occurs, producing immediate early (IE), delayed early (DE), and late (L) transcripts; immediate and delayed early genes have proposed roles in virus replication and nucleotide metabolism, while late gene products function in virion assembly and packaging [25,27]. From the nucleus, viral DNA is transported to the cytoplasm, where it is methylated [28,29] and large concatemers are formed [30]. Within the cytoplasm, viral DNA is encapsulated and remains either cell-associated (non-enveloped virions) or passes through the membrane acquiring an envelope [25].

While our understanding of FV3 and iridoviruses has substantially improved, the metabolic requirements for successful infection remain largely unexplored. Recently, it was reported that fatty acid synthesis [31], glucose [32,33], and glutamine metabolism [34] exert critical roles during *Iridoviridae* infection; however, no information is available regarding the role of purine metabolism. In this study, we explored the response of FV3 replication to exogenous application of components of the purine salvage pathway (adenine, adenosine, AMP, IMP, inosine, SAH, and SAM). We evaluated this in a FV3-permissive epithelial cell line [35,36], designated epithelioma papulosum cyprini, in an attempt to understand the role of purine salvage in FV3 infections. Contrary to other DNA viruses [13,14,15,17,18,19,20], we found that components of the purine salvage pathway, with the exception of SAH, increased FV3 replication.

## 2. Materials and Methods

### 2.1. Cell Culture, Reagents, and Virus

Epithelioma papulosum cyprini cells (EPC, American Type Culture Collection, ATCC No. CRL-2872) were maintained in Leibovitz’s L-15 media (L-15; Thermo Fisher Scientific, Waltham, MA, USA) containing 2.0 mM L-glutamine supplemented with 10% fetal bovine serum (FBS), 100 U/mL penicillin, 100 μg/mL of streptomycin, and 1.5 μg/mL of amphotericin B (Thermo Fisher Scientific, Waltham, MA, USA) at 20–25 °C.

Adenine, adenosine, adenosine 5′-monophosphate disodium salt (AMP), inosine, inosine 5′-monophosphate disodium salt hydrate (IMP), S-(5′-adenosyl)-L-homocysteine (SAH), and S-(5′-adenosyl)-L-methionine chloride dihydrochloride (SAM) were purchased from Sigma-Aldrich (Oakville, ON, Canada) and dissolved in 100% DMSO with the exception of SAM, which was dissolved in water, to prepare stock solutions (10 mM) for exogenous treatment. The concentration of DMSO in media did not exceed 0.5%. FV3 (ATCC No. VR-567) was propagated on confluent monolayers of EPC cells in 75cm^2^ flasks. EPC cells were infected at a multiplicity of infection (MOI) of 0.1 PFU/cell and harvested 5 days post infection when approximately 90% of the monolayer displayed cytopathic effects. Virus stocks underwent 3 freeze–thaw cycles to promote cell lysis and were partially purified by centrifugation at 4000 RPM for 5 min. Virus stocks were stored at −80 °C and titres were determined by plaque assay.

### 2.2. Relative Plaque Formation Assay

EPC cells were seeded using 10% L-15 media in 6-well plates to form confluent monolayers. Cells were concurrently infected with FV3 at an MOI of 0.1 PFU/cell and treated with either adenine, adenosine, AMP, IMP, inosine, SAH, or SAM at concentrations ranging from 0 to 30 μM in L-15 media supplemented with 1% FBS for 24 h. Inocula were removed, washed 3 times with 1× phosphate buffered saline (PBS, pH 7.2, Thermo Fisher Scientific, Waltham, MA, USA), and overlaid with 0.75% methylcellulose (Sigma-Aldrich, Oakville, ON, Canada) in L-15 supplemented with 1% FBS. After 3 days, plaques were counted using a Nikon Ts2R-FL inverted microscope (Nikon Canada Incorporated Instruments Division, Mississauga, ON, Canada) and plaque formation was calculated relative to the DMSO control. Statistical significance was assessed in GraphPad Prism 9 (GraphPad Software Incorporated, La Jolla, CA, USA) using a Kruskal–Wallis test followed by Dunn’s post hoc analysis. A *p*-value < 0.05 was considered significant and *n* represents the number of biological replicates analyzed.

### 2.3. Plaque Size Assay

EPCs were prepared as described for relative plaque formation. Cells were concurrently infected with FV3 at an MOI of 0.1 PFU/cell and treated with 20 μM of adenine, adenosine, AMP, IMP, inosine, SAH, or SAM for 4 h. Inocula were removed, monolayers were washed 3 times with 1× PBS (pH 7.2), and media was replaced with 1% L-15 containing 20 μM of the corresponding compound. After 24 h, cells were overlaid with 0.75% methylcellulose and images were taken 72 h post infection. Briefly, a series of images within a 250 mm^2^ area in the center of each well were taken using a EVOS XL Auto Imaging System (Thermo Fisher Scientific, Waltham, MA, USA), and Image J [37] was used to calculate the area of all plaques within this region. Statistical significance was assessed in GraphPad Prism 9 using a one-way ANOVA followed by Tukey’s multiple comparison’s test. A *p*-value < 0.05 was considered significant and *n* represents the number of biological replicates that were analyzed.

### 2.4. Cytotoxicity and Proliferation Assays

Cytotoxicity and proliferation in EPC cells were evaluated using the Cell Counting Kit 8 (WST-8/CCK8; Abcam, Toronto, ON, Canada) in 96-well plates seeded with 2 × 10^5^ cells and 1 × 10^5^ cells/well, respectively. Cells were treated with 30 μM of either adenine, adenosine, AMP, IMP, inosine, SAH, SAM, or a DMSO control in 1% L-15 media for 24 h at 20–25 °C. After 24 h, supernatant was removed and replaced with 1% L-15 lacking treatment compounds and incubated for an additional 48 h. Subsequently, 10 μL of WST-8 solution was added to each well, plates were incubated for 3 h at 20–25 °C and absorbance was measured at 460 nm using a SpectraMax M3 Multi-Mode Microplate Reader (Molecular Devices, LLC., San Jose, CA, USA).

## 3. Results

### 3.1. Exogenous Application of Adenine, Adenosine, AMP, IMP, Inosine, and SAM Stimulate FV3 Replication

**Figure 2:**
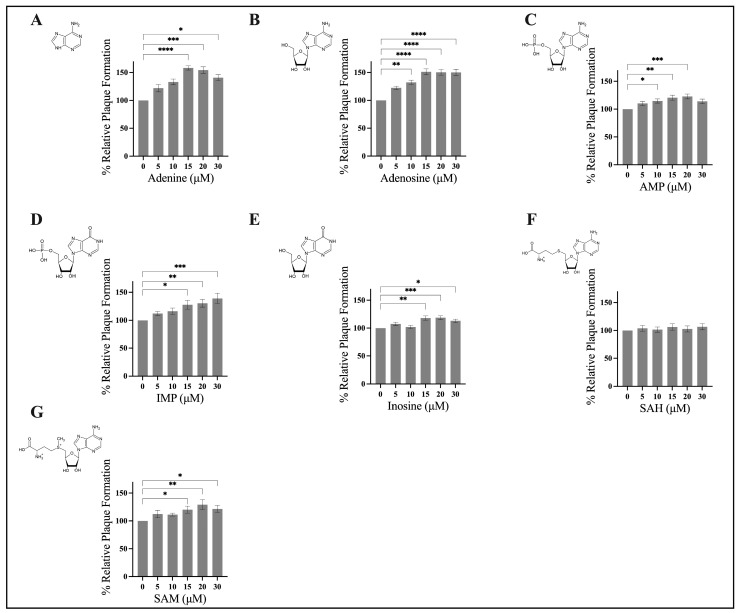
Components of the nucleotide salvage pathway increase frog virus 3 replication. EPC cells were concurrently infected with FV3 and treated with either exogenous (**A**) adenine, (**B**) adenosine, (**C**) AMP, (**D**) IMP, (**E**) inosine, (**F**) SAH, or (**G**) SAM at 0 to 30 μM for 24 h. After 24 h, cells were overlaid with methylcellulose, and plaque formation was assessed 72 h post infection. Data are presented as mean relative plaque formation (% of control) ±SEM. Statistical significance was evaluated using a Kruskal–Wallis test followed by Dunn’s post hoc analysis (*n* ≥ 3; * *p* ≤ 0.05, ** *p* ≤ 0.01, *** *p* ≤ 0.001, and **** *p* ≤ 0.0001).

To investigate whether components of the nucleotide salvage pathway have an impact on FV3 replication, we assessed FV3 replication using a relative plaque formation assay. EPC cells were contemporaneously infected with FV3 and exposed to either exogenous, adenine, adenosine, AMP, IMP, SAH, or SAM at concentrations ranging from 0 to 30 μM for 24 h. Initial evaluation revealed that adenine, adenosine, AMP, IMP, inosine, and SAM significantly increased FV3 replication in a dose-dependent manner (Figure 2). Specifically, we determined that adenine (Figure 2A) and adenosine (Figure 2B) had a maximal stimulatory effect at 15 μM, increasing FV3 replication by 58% and 51%, respectively. FV3 replication increased upon application of AMP (23%; Figure 2C), inosine (19%; Figure 2E), and SAM (29%; Figure 2G) at 20 μM. IMP (Figure 2D) increased FV3 replication by 39% at 30 μM, while SAH (Figure 2F) did not significantly alter FV3 replication at any concentration investigated. We conducted cell viability assays to exclude any cytotoxic or proliferative activity of these compounds as a reason for the observed increase in FV3 replication. We found that cytotoxicity and proliferation were unaltered (Table S1) by the compounds investigated, meaning the increase in FV3 replication was not due to any cytotoxic or proliferative impacts on the cells themselves. Accordingly, in order of effectiveness (most to least effective), adenine, adenosine, IMP, SAM, and inosine (Figure 2) stimulated rather than inhibited FV3 infection.

### 3.2. Application of Exogenous Adenine Increases FV3 Plaque Area

**Figure 3:**
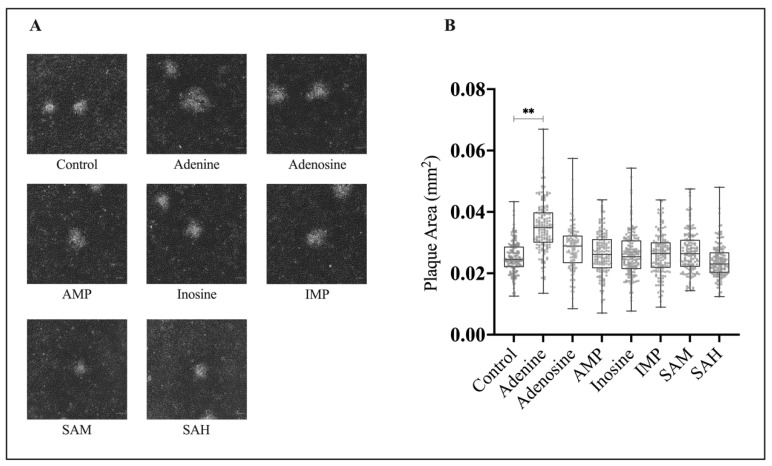
Adenine increases FV3 plaque area. (**A**) EPC cells were simultaneously infected with FV3 and either 20 μM of adenine, adenosine, AMP, inosine, IMP, SAM, or SAH and allowed to sit for 4 h. After 4 h, inocula were removed and replaced with 1% media containing 20 μM of the corresponding compound. After 24 h, cells were overlaid with methylcellulose and images were taken 72 h post infection. Images are representative of three independent experiments. (**B**) Plaque area was quantified using Image J, and data are presented as plaque area (mm²). Statistical significance was assessed using a one-way ANOVA followed by Tukey’s multiple comparison’s test (*n* = 3; ** *p* ≤ 0.01).

In addition to assessing viral fitness through relative plaque formation, we wanted to determine if application of these compounds produced any changes to plaque phenotype. To establish this, EPC cells were concurrently infected with FV3 at an MOI of 0.1 PFU/cell and 20 μM of adenine, adenosine, AMP, inosine, IMP, SAM, or SAH. FV3 plaque area analysis revealed that plaques formed in the presence of adenine (M = 0.034 mm^2^) were 40% larger than those of the control (M = 0.025 mm^2^; Figure 3). There was an increase in plaque area in the presence of adenosine (M = 0.029 mm^2^); however, this was not significant. Similarly, there was no significant difference in the plaque area of the control (M = 0.025 mm^2^) when compared to plaques that formed in the presence of AMP (0.027 mm^2^), inosine (M = 0.025 mm^2^), IMP (0.026 mm^2^), SAM (0.027 mm^2^), or SAH (0.024 mm^2^; Figure 3).

### 3.3. Pre-Treatment Assays

**Figure 4:**
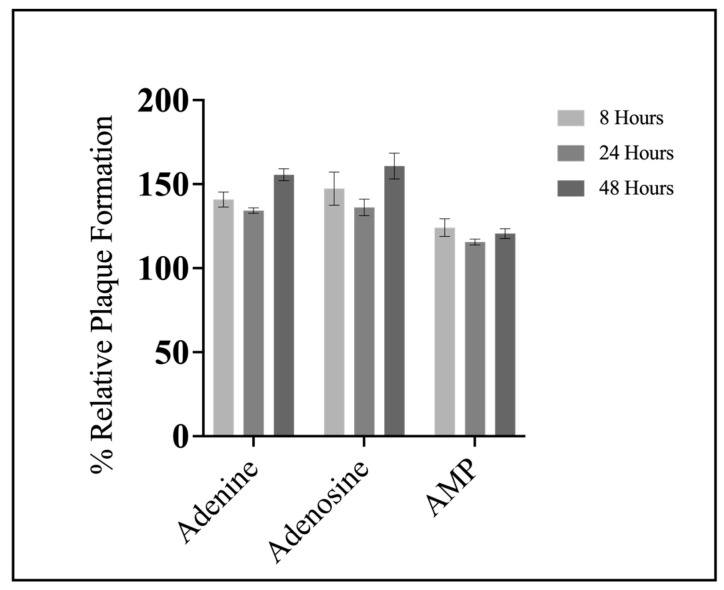
Pre-treatment with compounds of the nucleotide salvage pathway is sufficient to increase FV3 replication. EPC cells were exposed to 20 μM of either adenine, adenosine, or AMP for 8, 24, or 48 h. After the designated time, compounds were removed, and cells were infected with FV3 for 24 h. Twenty-four hours post infection, inocula were removed, cells were overlaid with methylcellulose, and plaque formation was assessed 72 h post infection. Data are presented as mean relative plaque formation (% of control) ±SEM. Statistical significance was evaluated using a two-way ANOVA followed by Tukey post hoc analysis (*n* = 3).

Based on the findings that all compounds except SAH significantly increased FV3 replication, but only adenine significantly increased plaque area, we wanted to assess if concurrent treatment was required for increased FV3 replication or if pre-treatment was sufficient to induce a similar response. EPC cells were pre-treated with 20 μM of either adenine, adenosine, or AMP for 8, 24, or 48 h, after which, the adenylates were removed and cells were infected with FV3 at 0.1 PFU/cell for 24 h. FV3 replication increased upon 8, 24, and 48 h pre-treatment with adenine, adenosine, and AMP; the increase observed upon pre-treatment (Figure 4) are comparable to those of concurrent application (Figure 3). The timing of pre-treatment had no significant impact on FV3 replication. For example, after 8 h pre-exposure to adenine, FV3 replication increased by 41%, while 48 h pre-exposure increased FV3 replication by 55% (Figure 4). Overall, 8 h pre-exposure to adenine, adenosine, or AMP was sufficient to increase FV3 replication, and this increase in replication was sustained in cells pre-treated up to 48 h prior to infection (Figure 4).

## 4. Discussion

In this study, we found that exogenous application of components of the nucleotide salvage pathway including adenine, adenosine, adenosine 5′-monophosphate (AMP), inosine 5′-monophosphate (IMP), inosine, and S-adenosyl-L-methionine (SAM) increased frog virus 3 (FV3) replication. The finding that purines within the nucleotide salvage pathway increased FV3 replication is, to our knowledge, the first to be reported in DNA viruses. Sabariegos et al. [13] found that HCV replication was reduced due to altered NS5B RNA polymerase activity that could be attributed to changes in nucleoside di- and triphosphate levels. Specifically, application of ribonucleosides (adenosine, guanosine, uridine, and cytidines) increased intracellular nucleoside diphosphates (ADP, CDP, UDP, and GDP) and their corresponding triphosphates. Moreover, metabolic profiling of cells infected with influenza virus revealed decreased ATP concentrations, but increased ADP and AMP concentrations over the course of infection [37]. Mechanistically, it is unclear how the addition of these molecules produces a response in FV3. It is plausible that the exogenously applied purines act as substrates that can be interconverted to replenish purine pools that are normally depleted during infection. Future research should examine changes in intracellular metabolites and track the formation of metabolic byproducts that may be produced as a result of exogenously applied purines.

Infection with ranaviruses produces a robust host inflammatory response. In EPC cells, ranaviral infections are associated with upregulated levels of pro-inflammatory cytokines, TNF-α and IL-1β [36], while ranaviral infection of fathead minnow (FHM) cells produces marked increases in the transcription of interleukins IL-1β, IL-8, IL-17C, and IL-12 [38]. Ranaviruses encode viral homologs of dUTPase, DNA cytosine methyltransferase (DMTase), and a tumor necrosis factor (TNF) receptor, which are believed to circumvent the protection that is conferred by the upregulation of pro-inflammatory cytokines and interferons [39,40]. Adenosine is generally considered to act as a protective and homeostatic regulator for cellular immunity and inflammation. It was demonstrated that adenosine acts to modulate TNF-α signalling through suppression of the NF-κB pathway [41,42,43], which inhibits the expression of IL-8 and IL-1β [42,43,44]. In addition, research showed that inosine acts to inhibit the production of TNF-α, IL-1, IL-12, macrophage inflammatory protein 1α, IFNγ, and IL-8 in an endotoxemic mouse model [45] and human epithelial cells [46]. Given the fact that virally infected cells generate an immunosuppressive environment [47], the application of exogenous purines, particularly adenosine, may interfere with the production of cytokines and interferons. That is, the application of exogenously applied purines accompanied by the immune evasion strategies utilized by ranaviruses may create an immunosuppressive environment that facilitates increased viral replication.

Previous reports outlining the potent antiviral capacity of purines emphasized high micromolar to millimolar concentrations [13,14,16,17,18,20]. These concentration ranges may be useful for establishing the antiviral capacity of purines, but may not be physiologically relevant. In mammals, extracellular purine-free bases and nucleosides exist in a range of 0.4–6 μM dependent on species and tissue type; extracellular values are usually lower than intracellular values [48,49]. In HeLa cells, purine triphosphates (ATP and GTP) exist at 0.02–2.74 nmol/million cells, while purine monophosphates (AMP and GMP) exist at 0.17–0.23 nmol/million cells [50]. The purine composition of EPC cells has not been reported; however, it is evident that this study employs considerably lower concentrations than previous investigations [13,14,17], which are likely more biologically relevant, but this remains to be confirmed. Alternatively, small molecules are known to produce biphasic dose responses. As such, purines may result in stimulation of viral replication at low doses (as observed in this study), but result in inhibition at higher doses [13,14,15,17,18,19,20].

In conjunction with our assessment of FV3 replication to exogenous purines through relative plaque formation, we further evaluated plaque area. The findings for plaque area are consistent with relative plaque formation; an increase in relative plaque formation (Figure 2) correlates with an increase in plaque area (Figure 3). Interestingly, application of adenine and adenosine at 20 μM increased FV3 replication by 55% and 50%, respectively (Figure 2); however, only application of adenine produced a statistically significant increase in plaque area (Figure 3). There are numerous determinants of plaque size, including virus replication, cell-to-cell spread, viral egress, and cellular immune responses to viral infection [51,52,53]. As such, the observed significant increase in FV3 plaque area upon exposure to adenine, but not adenosine or the other compounds investigated, may indicate that the stimulatory effect on FV3 replication by adenine is at least in part mediated by a mechanism that is independent from the other compounds investigated.

The finding that components of the purine salvage pathway increased FV3 replication may represent an important finding for other members of the *Ranavirus* genus. In the purine salvage pathway, purine nucleoside phosphorylase (PNP) is responsible for the interconversion of nucleosides to their corresponding free bases (Figure 1) [54,55]. Genomic analysis revealed that grouper iridovirus (GIV) encodes a homologue of mammalian PNPs designated givPNP [54,55]. Reports are conflicting regarding its enzymatic activity with Ting et al. [55], suggesting that givPNP can use guanosine, inosine, and adenosine as substrates, while Kang et al. [54] note that substrate specificity is limited to inosine and guanosine. The presence of PNP in GIV may indicate that GIV evolved a mechanism to acquire an adequate amount of purines that would otherwise not be provided by its hosts [54,55]. The existence of a virally encoded PNP in GIV and our finding that exogenously applied purines increase FV3 replication clearly highlight the importance of purine availability during ranaviral infection. As such, targeting purine metabolism may represent a previously unexplored strategy for understanding ranaviral infection strategies.

In conclusion, cellular purines are a class of molecules pivotal for cellular processes ranging from metabolism to cell signaling. In this study, we describe the response of FV3 to exogenous application of compounds found within the purine salvage pathway. The application of exogenous adenine, adenosine, AMP, inosine, IMP, and SAM increased viral production. The finding that purines enhance FV3 replication is the first to our knowledge to be reported in DNA viruses. Although the mechanism of action is unclear, these results may add key insights to the importance of nucleotide metabolism during *Iridoviridae* infections.

## Data Availability

Data is contained within the article or supplementary material.

## Supplementary Materials

The following can be downloaded at: https://www.mdpi.com/article/10.3390/v15081716/s1, Table S1: The cytotoxic (CC_50_) and proliferative (EC_50_) concentrations of compounds of the purine salvage pathway in EPC cells after 24-h exposure.
