# Supplementary material for: Components of the Nucleotide Salvage Pathway Increase Frog Virus 3 (FV3) Replication
Source: Viruses. 2023 Aug 10;15(8):1716. doi: 10.3390/v15081716 (PMC10460048; doi:10.3390/v15081716)
Supplement: Supplementary file 1 [file viruses-15-01716-s001.zip]

| Compound Name | CC_50_^a^ | EC_50_^a^ |
| --- | --- | --- |
| adenine | >30 | >30 |
| adenosine | >30 | >30 |
| adenosine 5’-monophosphate | >30 | >30 |
| inosine | >30 | >30 |
| inosine 5’-monophosphate disodium salt hydrate | >30 | >30 |
| S-(5′-adenosyl)-L-homocysteine | >30 | >30 |
| S-(5′-adenosyl)-L-methionine chloride dihydrochloride | >30 | >30 |

Supplementary Table S1. The cytotoxic (CC_50_) and proliferative (EC_50_) concentrations of compounds of the purine salvage pathway in EPC cells after 24-hour exposure.

^a^All values are in µM, based on 2 independent experiments each comprised of three replicates.
